# Effects of Salicylic Acid on Heavy Metal Resistance in Eukaryotic Algae and Its Mechanisms

**DOI:** 10.3390/ijerph192013415

**Published:** 2022-10-17

**Authors:** Tingting Zhang, Mei Shi, Hao Yan, Cheng Li

**Affiliations:** School of Life Sciences, Anhui Normal University, Wuhu 241000, China

**Keywords:** Cd^2+^ pollution, eutrophication, salicylic acid, *S. obliquus*, *C. pyrenoidosa*

## Abstract

Heavy metal pollution and water eutrophication are still the main issues to be solved in the environmental field. To find a biological control method for Cd^2+^-contaminated water or combined eutrophication and Cd^2+^ pollution water, the effects of salicylic acid on heavy metal Cd^2+^ resistance in eukaryotic algae *Scenedesmus obliquus* and *Chlorella pyrenoidosa* and its mechanisms were studied. The results showed that the inhibition rates of 3.0 mg/L Cd^2+^ stress group at 96 h were 67.0% on *S. obliquus* and 61.4% on *C. pyrenoidosa* and their uptake of Cd^2+^ was 0.31 mg/g and 0.35 mg/g, respectively. When adding the different concentrations of salicylic acid while stressed by 3.0 mg/L Cd^2+^, the hormesis phenomenon of low dose stimulation and high dose inhibition could be seen, and the inhibition rates of 30 mg/L~90 mg/L salicylic acid addition groups were significantly lower than those of the Cd^2+^ stress group alone, which were statistically significant (*p* < 0.05) and the absorption of Cd^2+^ was dramatically improved. Except for the 120 mg/L salicylic acid addition group, the chlorophyll fluorescence parameters (Fv/Fm and YII), glutathione peroxidase (GSH-Px) and glutathione-S-transferase (GST) activities of all the other concentration groups were significantly higher than those of the Cd^2+^ stress group alone, *p* < 0.05.; the algal cell morphology in low concentration groups (30 mg/L and 60 mg/L) was also less damaged than those in the Cd^2+^ stress group alone. These indicate that the low concentrations of salicylic acid can counteract or protect the algal cells from Cd^2+^ attack, the mechanisms, on the one hand, might be related to the chelation of heavy metals by salicylic acid, resulting in the decrease of the toxicity of Cd^2+^; on the other hand, low concentrations of salicylic acid can stimulate the growth of these two algae, improve their photosynthetic efficiency and antioxidant capacity, as well as maintain the relative integrity of algal morphological structure.

## 1. Introduction

Since the 1980s, with the rapid development of China’s industrial and agricultural production, the problem of contamination in rivers, lakes and reservoirs has become increasingly prominent. Under a series of measures, such as the National Science and Technology Major Project on Water Pollution Control and Management, the level of water contamination has been greatly attenuated, and eutrophication has been significantly alleviated. However, the problems are still relatively prominent, especially severe heavy metal pollution. Huang et al. monitored 138 lakes and reservoirs in 2017 and found that 38 lakes and reservoirs (up to 27.5%) had heavy metal pollutants, and the concentration was higher than the standard of inferior five waters [1]. Some lakes and reservoirs still had severe eutrophication problems. Moreover, eutrophication and heavy metal pollutants exist in many water bodies simultaneously, with the Cd^2+^ topping all heavy metal contaminants [2,3,4].

The Cd^2+^ pollution and eutrophication of water cause great harm to plants and animals in aquatic ecosystems and can lead to deterioration of water quality, imbalance of ecosystem balance and reduction of biodiversity [5,6]. In some areas, the Cd^2+^ pollution of rice is serious, the samples with Cd^2+^ exceeding the standard even reached more than 70% [6]. Cd^2+^ and cyanobacterial toxins secreted by cyanobacteria can be enriched in the food chain, long-term drinking and consumption of water and food contaminated with Ca^2+^ and cyanobacterial toxins will cause them to accumulate in human body, posing a great threat to human health. Ititai disease is the most famous disease caused by Cd^2+^, Cd^2+^ can also seriously affect human nerves, liver, kidneys, blood and even cause cancer [7,8]. *Microcystis*, the most common algae in water blooms, can secrete hepatotoxins, neurotoxins, cytotoxins and dermatoxins. In addition to the fact that hepatotoxins are known to induce liver cancer, these toxins can damage the body in a number of ways [9].

Therefore, controlling water eutrophication and heavy metal pollution is still an important task in the environmental field. In the control and treatment studies of water bloom in eutrophic water bodies, the suppressive influence of allelochemical substances secreted by aquatic plants on water bloom has been widely concerned [10,11]. The inhibition of allelopathic substances on algae is usually selective in terms of which phenolic acids are the most studied [12]. Salicylic acid is a phenolic acid widely found in plants and is also an endogenous hormone, which has been found to have an excellent inhibition function on cyanobacteria [13,14,15,16]. Our research group has systematically studied the algae-inhibiting effects of various phenolic acids, especially salicylic acid [16]. Surprisingly, salicylic acid not only inhibits cyanobacteria, such as *Microcystis aeruginosa*, and does not cause the increase of cyanobacterial toxins in water [13,16]; but salicylic acid has obvious low-promoting and high-inhibitory effects on eukaryotic green algae *S. obliquus* and *C. pyrenoidosa*. Therefore, the concentration of salicylic acid that can effectively inhibit cyanobacteria, often promoting the growth of these two types of green algae [17,18].

There are many methods for removing heavy metals from water, such as ion exchange, precipitation, chemical extraction, etc. However, these methods are often ineffective and expensive when practically applied to large projects [19]. Therefore, the use of algae to remove heavy metals is considered to be an economical and environmentally friendly biological method, because algae have simple nutrition types, large volume specific surface area, strong metal removal ability and easy regeneration of algae and metal can be recycled after being absorbed by algae and resource utilization [20,21].

In eutrophic water, there are usually mixed algae, such as cyanobacteria and green algae, while the more common and concerned eukaryotic green algae are *C. pyrenoidosa* and *S. obliquus*, the latter of which is the indicator organism of A-type mesosaprobic zone in eutrophic water [22,23]. At present, the resistance of these two kinds of algae to heavy metals has been reported [24,25]. Coincidentally, salicylic acid, as a mysterious signaling molecule in plants, has been extensively studied for its defense against cadmium stress in plants [26]. Therefore, while using salicylic acid to inhibit cyanobacteria, if there is heavy metal pollution, the effect of salicylic acid on the heavy metal resistance of eukaryotic algae in this water is worthy of further investigation. So, in this study, taking the eukaryotic algae *S. obliquus* and *C. pyrenoidosa* as the test objects, the effects of salicylic acid on their growth under Cd^2+^ stress and the mechanisms of the cell morphology, photosynthetic parameters, antioxidant enzyme activity and enrichment capacity of Cd^2+^ were carried out in order to find a method to inhibit cyanobacteria and absorb heavy metals in water at the same time and provide new ideas and experimental basis for the biological removal of heavy metal pollutant as well as heavy metal pollutant in eutrophic water.

## 2. Materials and Methods

### 2.1. Materials and Instruments

*S. obliquussa* and *C. pyrenoidsa* were purchased from the Institute of Hydrobiology, Chinese Academy of Sciences, and cultured in HB-4 medium; salicylic acid and CdCl_2_ were purchased from Sinopharm Chemical Reagent Co., Ltd., Shanghai, China; 94% propidium iodide (PI) and 97% fluorescein diacetate (FDA) were purchased from Shanghai Aladdin Biochemical Technology Co., Ltd., Shanghai, China; GSH-Px and GST activity assay kits were purchased from Nanjing Jiancheng Biological Engineering Research Institute.

The Lainde LD-96A automatic enzyme-linked immunosorbent assay systems are the products of Shandong Lainde Intelligent Technology Co., Ltd., Shandong, China; the scanning electron microscope (SEM) is the JEOL product from Japan; AA320 atomic absorption spectrophotometer is the product of Shanghai Precision Instrument Co., Ltd., Shanghai, China; and the FMS-2 Portable Pulse Modulated Fluorometer is the product of the British Hansatech Company, Shanghai, China.

### 2.2. Experimental Methods

#### 2.2.1. Algal Culture

One week before the experiment, both *S*. *obliquus* and *C*. *pyrenoidosa* were expanded and cultured individually. Under aseptic conditions, 400 mL of HB-4 medium was added to a sterilized 1000 mL triangular conical flask and the algal species of *S*. *obliquus* and *C*. *pyrenoidosa* were added, shaken well and cultivated in a light incubator. The culture conditions were illuminance 4000 lx, light-dark ratio 12 h:12 h, temperature (25 ± 1) °C and a pH 7.0. The conical flask was shaken 3–4 times a day, and the appropriate amount of fresh medium was added regularly for seven days to make the algae cells enter the logarithmic phase [16].

#### 2.2.2. Effects of Salicylic Acid on Biomass of *S. obliquus* and *C. pyrenoidosa* under Cd^2+^ Stress

*S. obliquus* and *C. pyrenoidosa* at the logarithmic growth stage were poured into 500 mL conical flasks at the logarithmic growth stage and then added into a medium to make the initial density of algal cells 5.5~5.7 × 10^6^ cells/mL, separately. The volume was finally maintained at 300 mL. The concentration of Cd^2+^ in the Erlenmeyer flask was 3.0 mg/L, and the concentrations of salicylic acid were 30, 60, 90 and 120 mg/L, respectively. The control groups without Cd^2+^ and salicylic acid (ck1) and the control group with only Cd^2+^ without salicylic acid (ck2) were set up and each group (three replicates) was incubated under the same conditions of 1.2.1. Cell counts were performed every 24 h [27].

#### 2.2.3. Effects of Salicylic Acid on the Chlorophyll Fluorescence Parameters of *S. obliquus* and *C. pyrenoidosa* under Cd^2+^ Stress

Chlorophyll fluorescence parameters were determined using an FMS-2 portable pulse-modulated fluorometer. Every 24 h, 2 mL of each sample solution from experiment in Section 2.2.2 was taken and dark-adapted for 20 min at a room temperature of 20 °C. The fixed fluorescence yield of chlorophyll (F_0_) was determined by measuring the light beam and the maximum fluorescence yield (Fm) and actual light energy conversion efficiency (YII) were measured with a saturating light pulse. Then the maximum quantum yield of photosystem II (Fv/Fm) could be calculated from F_0_ and Fm: Fv/Fm = (Fm − F_0_)/Fm [28].

#### 2.2.4. Effects of Salicylic Acid on Oxidase Activity of *S. obliquus* and *C. pyrenoidosa* under Cd^2+^ Stress

Algal solution 10 mL was taken from the conical flasks of the experiment in Section 2.2.2 every 24 h and centrifuged (4000 rpm for 15 min). Then the supernatant was removed, 1 mL of PBS buffer was added to the algal mud and the mixture was put in a −20 °C refrigerator overnight. The cell walls were broken by repeated freeze-thawing and the supernatant was collected after centrifugation at 12,000 rpm for 5 min at 4 °C [29]. GSH-Px and GST activities were determined according to the instructions of the corresponding enzyme activity assay kits, respectively. The unit of enzyme activity was specified as a drop of 1 μmol/L GSH concentration in the reaction system per mg of protein per minute at 37 °C after the deduction of the non-enzymatic reaction.

#### 2.2.5. Effects of Salicylic Acid on Cell Morphology of *S. obliquus* and *C. pyrenoidosa* under Cd^2+^ Stress

The morphology of algal cells was observed by SEM. On the 96th hour of the experiment in Section 2.2.2, the algal liquid was collected, centrifuged at 2500 rpm to harvest the algal cells, washed 2–3 times with a sterile clean medium, fixed in 2.5% glutaraldehyde for 2 h and then buffered with freshly prepared 0.1 mol/L PBS. The solution was rinsed three times and finally dehydrated with a series of ethanols, replaced with isoamyl acetate, dried at the critical point of CO_2_, metal sprayed and observed by scanning electron microscope [27].

#### 2.2.6. Effects of Salicylic Acid on the Absorption of Cd^2+^ by *S. obliquus* and *C. pyrenoidosa*

At the 96th hour of the experiment in Section 2.2.2, 50 mL of both *S. obliquus* and *C. pyrenoidosa* were taken, washed three times with 15 µg/mL NaHCO_3_ and distilled water separately, dried, weighed and digested. The content of Cd^2+^ was determined using a J-8000 atomic absorption spectrophotometer [27,28]. 

### 2.3. Data Processing and Analysis

Data were processed using Microsoft excel software and the inhibition rate was calculated using the formula IR(%) = (1 − N/N_0_) × 100, where N and N_0_ were the algal density of the experimental group and the control group, respectively. Statistical analysis was performed using SPSS 22.0 analysis software and the one-way ANOVA tested the significance of algae inhibition in each experimental group; multiple comparisons between groups were performed using the least significant difference (LSD) method, with *p* < 0.05 indicating significant differences and *p* < 0.01 indicating highly significant differences.

## 3. Results

### 3.1. Effects of Salicylic Acid on the Biomass of S. obliquus and C. pyrenoidosa under Cd^2+^ Stress

**Figure 1:**
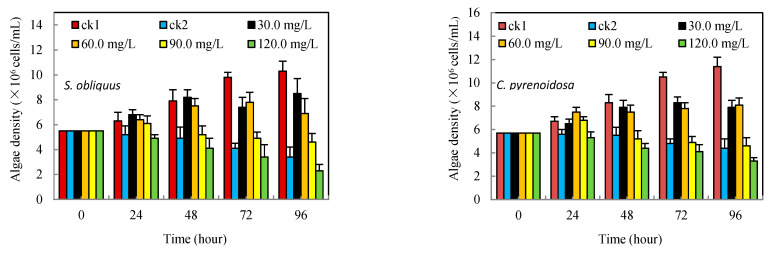
Effects of salicylic acid on the biomass of *S. obliquus* and *C. pyrenoidosa* under Cd^2+^ stress.

As can be seen from Figure 1, ck2 was the control group containing only Cd^2+^. Although the two algae strains were resistant to it, their algal density kept decreasing from the 24th to 96th h after the beginning of the experiment. Compared with the control group in the same period, the inhibitory rates were 67.0% and 61.4%, respectively (see Table 1), while the density of algae in the salicylic acid-added groups was significantly different. The low concentration of salicylic acid (30–60 mg/L) showed a slight increase in the density of *S. obliquus* at the 24th and even 48th h (30 mg/L). The density of *C. pyrenoidosa* also showed a mild elevation at 24th h for the 60–90 mg/L concentration group, but the inhibition rate became positive with time and the further increment of salicylic acid concentration. Almost all concentration salicylic acid-added groups had lower inhibitory rates than ck2 at any time, except for the 120 mg/L groups, which suggested that a particular concentration of salicylic acid was beneficial to improve the resistance of the two eukaryotic algae to Cd^2+^ stress.

### 3.2. Effects of Salicylic Acid on Chlorophyll Fluorescence Parameters of S. obliquus and C. pyrenoidosa under Cd^2+^ Stress

**Figure 2:**
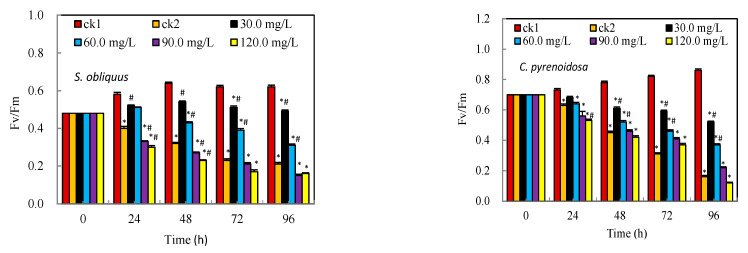

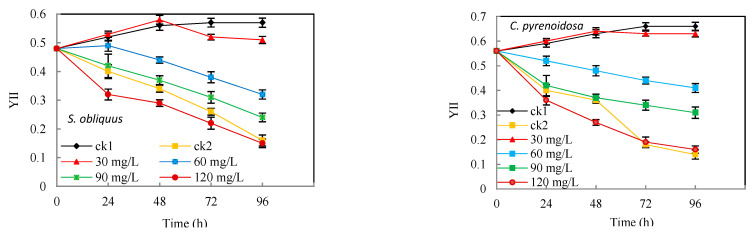
Effects of salicylic acid on the Chlorophyll fluorescence parameters of *S. obliquus* and *C. pyrenoidosa* under Cd^2+^ stress. Note: * means there is statistical significance compared with the ck1 group at the same period, *p* < 0.05; # means there is a statistical significance compared with the ck2 group at the same period, *p* < 0.05.

Fv/Fm reflects the maximum light energy conversion efficiency of photosystem II, while YII reflects the actual light energy conversion efficiency of photosystem II. As shown in Figure 2, the Fv/Fm and YII of *S. obliquus* and *C. pyrenoidosa* showed a significant descent (*p* < 0.05) in the first 24 h under Cd^2+^ stress alone and the decline became more pronounced with time. While the Fv/Fm of both *S. obliquus* and *C. pyrenoidosa* were lower than that of the control group (ck1) at the same time, the group with 30–60 mg/L salicylic acid addition was consistently higher than the Cd^2+^ alone group (ck2), *p* < 0.05. It indicates that under the stress of Cd^2+^ and at relatively high concentrations of salicylic acid, *S. obliquus* and *C. pyrenoidosa* occurred photoinhibition or some degree of the disruption of photosystem II structure. However, the low concentrations of salicylic acid evidently counteracted or prevented the algal cells from Cd^2+^ attack at some degree, the YII was even higher than ck1 at 24 h and 48 h in the 30 mg/L group.

### 3.3. Effects of Salicylic Acid on Oxidase Activity of S. obliquus and C. pyrenoidosa under Cd^2+^ Stress

**Figure 3:**
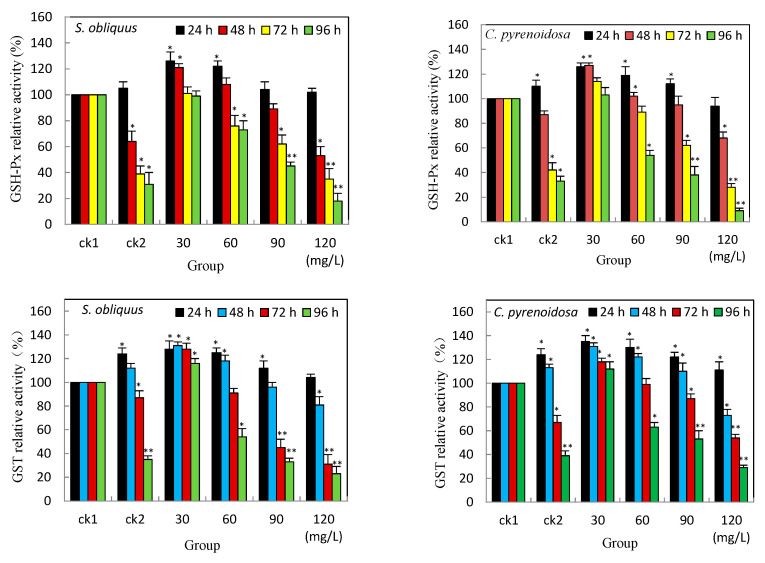
Effects of salicylic acid on the GSH-Px and GST activities of *S. obliquus* and *C. pyrenoidosa* under Cd^2+^ stress. Note: * means there is statistical significance compared with the ck1 group at the same period, *p* < 0.05; ** means compared with the ck1 group at the same period, *p* < 0.01.

The activities of both GSH-Px and GST have unique roles in resistance to heavy metal oxidative stress. As shown in Figure 3, both GSH-Px and GST activity elevated at the early stage (24 h) under Cd^2+^ stress alone (ck2), and the GSH-Px activity of *C. pyrenoidosa* was higher than that of *S. obliquus,* it was statistically significant (*p* < 0.05), compared with the control group (ck1); the elevation of GST activity was statistically significant in both *S. obliquus* and *C. pyrenoidosa* at 24 h in ck2 group. All groups with salicylic acid added under Cd^2+^ stress both GSH-Px and GST activity increased at the beginning of 24 h. The elevation was significant (*p* < 0.05) in the low concentration group at 30 mg/L but not in the highest concentration group compared with ck1. The increase in the activity of GST was more pronounced than that of GSH-Px in all groups.

### 3.4. Effects of Salicylic Acid on Cell Morphology of S. obliquus and C. pyrenoidosa under Cd^2+^ Stress

**Figure 4:**
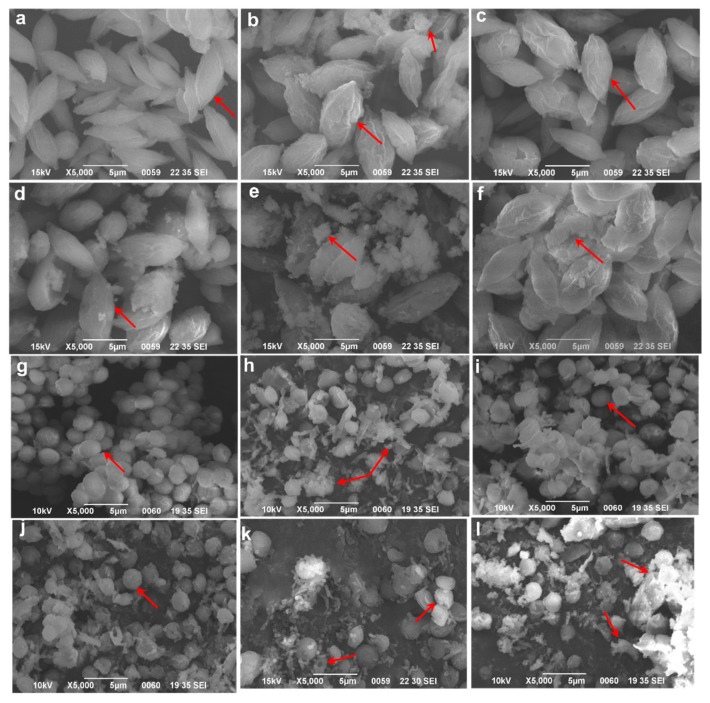
Effects of different concentrations of salicylic acid on the surface structures of *S. obliquus* (**a**–**f**) and *C. pyrenoidosa* (**g**–**l**) under Cd^2+^ stress after 96 h (bar = 5 μm) Note: (**a**,**k**): ck1; (**b**,**h**): ck2; (**c**,**i**): 30 mg/L; (**d**,**j**): 60 mg/L; (**e**,**k**): 90 mg/L; (**f**,**l**): 120 mg/L. All arrows in the figure indicate the phenomena described in the paragraph.

It can be found from Figure 4 that after 96 h of the experiment, under conditions without Cd^2+^ (ck1), the algal cells of both *S. obliquus* and *C. pyrenoidosa* were distinct individually with complete morphologies and plump with a smooth surface. In the *S. obliquus* group containing only Cd^2+^ (ck2) under the same magnification, the algal cells showed noticeable changes when swollen with cells being white and even flocculent, indicating that some cell walls or plasma membranes had been ruptured, and the cellular components ooze. However, under Cd^2+^ stress, the cell damage of *S. obliquus* in the low-concentration salicylic acid added groups (30–60 mg/L) were much less damaged than that of the ck2, although there were also significant changes compared with ck1. It was only with the increase in salicylic acid concentration that the algal cell destruction intensified. The individual cells were indistinguishable, almost all algal cells turned white and there was a large amount of flocculent material on their surface and around them. The phenomenon of multiple cells and their cell fragments adhering into clusters was observed under SEM. In the *C. pyrenoidea* group under pure Cd^2+^-stress (ck2), the cell swelling was relatively insignificant, but the rupture was significantly more than that in the *S. obliquus* ck2 group. The algal cells in the low-concentration salicylic acid addition group (30–60 mg/L, especially 30 mg/L) were less damaged, mostly spherical and smooth; with the increase of salicylic acid concentration (90–120 mg/L), the cell rupture was aggravated and a large amount of cell debris and white flocs appeared but the difference was not significant compared with its ck2 group.

### 3.5. Effects of Salicylic Acid on the Absorption of Cd^2+^ by S. obliquus and C. pyrenoidosa

**Figure 5:**
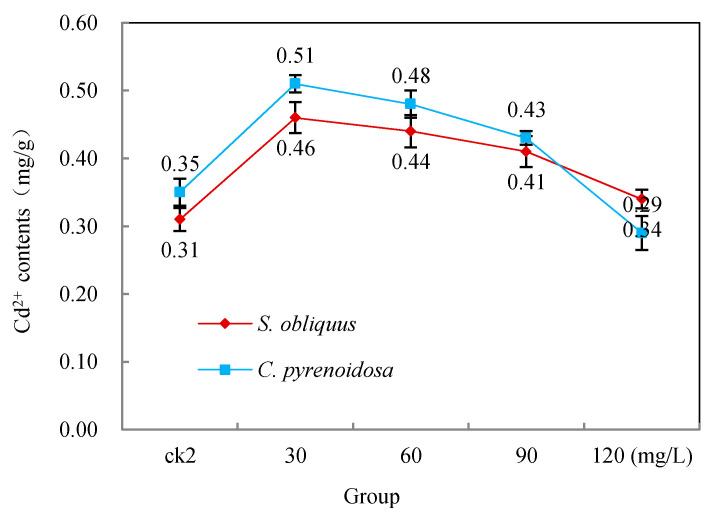
Effects of salicylic acid on the Cd^2+^ uptake of *S. obliquus* and *C. pyrenoidosa* after 96 h.

Figure 5 shows the effect of salicylic acid additions on Cd^2+^ uptake by both *S. obliquus* and *C. pyrenoidosa*. It can be seen from ck2 in Figure 5 that both *S. obliquus* and *C. pyrenoidosa* had some uptake capacity for Cd^2+^. However, the Cd^2+^ uptake in the group with low salicylic acid addition (30–60 mg/L) was significantly higher than that in ck2, which was statistically significant (*p* < 0.05). When salicylic acid was added at 90 mg/L, the Cd^2+^ uptake was still higher than that of ck2, although it was not statistically significant. Only the 120 mg/L group was lower than ck2, but it was not statistically significant.

## 4. Discussion

As a phenolic acid allelochemical, salicylic acid is one of the most reported and effective cyanobacterial inhibitors [13,14,15,16]. In addition, salicylic acid has been reported in previous papers as some green algae promoter that inhibits the growth of cyanobacteria within a certain concentration range [17,18]. The low concentration (30–60 mg/L) of salicylic acid set in this study is exactly the concentration reported in much of the previous literature that can significantly inhibit the growth of cyanobacteria [13,14,15,16]. Liu et al. reported that 25~40 mg/L salicylic acid exhibited a significant inhibitory effect on the growth of the cyanobacteria *Synechococcus*, with an inhibition rate of about 70% [15]. Hu et al. reported that salicylic acid had a significant inhibitory effect on *M. aeruginosa* when the concentration of salicylic acid was 20 mg/L and the inhibitory effect became more pronounced as the concentration increased, while 120 mg/L salicylic acid was used for 2 days and the maximum inhibition rate almost reached 100% [14]. In this study, even in the presence of 3.0 mg/L Cd^2+^, 30 mg/L salicylic acid was able to resist Cd^2+^ stress and stimulate the growth of *C. pyrenoidosa* in 48 h. Although there was a slight inhibitory effect on *S. obliquus* at this concentration and time, it was not significant and there was a very significant difference compared with the pure Cd^2+^ stress group (ck2). Therefore, the results of this study demonstrate the stimulating properties of salicylic acid on certain eukaryotic green algae, which are consistent with previous studies [17,18]. You et al. reported that the density of *S. obliquus* reached the maximum value in the 20 mg/L salicylic acid addition group, and the biomass increased by 34.47% compared with the control group [17]. Wu et al. added different concentrations of salicylic acid to two Chlorella strains, namely *C. vulgaris* ZF strain and FACHB31 strain, and found that 10 mg/L salicylic acid was able to increase the biomass of the former [18].Therefore, adding a specific range of low-concentration salicylic acid to Cd^2+^-contaminated water, alone or in combination with eutrophication and Cd^2+^ pollution, was able to significantly improve Cd^2+^ absorption or elimination. Especially in eutrophic water, supplementing a certain amount of salicylic acid can effectively inhibit cyanobacteria on the one hand, and on the other hand, can promote the absorption of Cd^2+^ by eukaryotic algae, which is twice the result with half the effort. However, different algae or even different algal strains of the same algae have different susceptibility to salicylic acid [18], and further research is needed in the future.

Other biological methods, such as bacteria, fungi, etc., are also known to be effective in removing heavy metals [30]. However, compared with eukaryotic green algae, these methods may have secondary pollution, as a large number of bacteria are difficult to remove and even if they are not pathogenic bacteria, they may become opportunistic pathogenic bacteria under certain conditions. However, eukaryotic algae themselves exist in eutrophic water bodies. By adding appropriate plant hormones, their resistance to heavy metals can be greatly improved and they are easier to recover than bacteria, which has certain economic and practical significance [18].

The cell wall composition of different algae varies greatly. Green algae have strong metal ion absorption properties due to their cell walls containing many celluloses and high protein content, forming amino, carboxyl, sulfate and hydroxyl groups. Most of these groups can adsorb metals, such as oxygen in the carboxyl group (-COOH) or hydroxyl group (-OH), since unbonded electrons can cooperate with the empty orbitals of metal ions to form coordinate bonds [21].

The accumulation of metals in algal cells is also a tolerance mechanism, with Cd^2+^ being chelated into a non-toxic form. It has been shown that certain heavy metals are deposited on cell surface vacuoles, while Cd^2+^ is exclusively observed in chloroplasts. Salicylic acid molecules contain not only carboxyl groups but also hydroxyl groups, and all these organic functional groups have the property of chelating heavy metals [29]. By forming complexes with metals, the concentration of metals in the free ionic state can be reduced, decreasing the toxicity of metals. In addition, the low concentrations of salicylic acid can augment algal biomass, while many algae can also secrete polyphenolic compounds that can bind heavy metal ions. Some of these polyphenols also act as metal chelators in the cell walls and the algal cells. Salicylic acid, as a small molecule phenolic acid, can also enter the cell to play the role of metal chelator under certain conditions [21,26], so the addition of salicylic acid enhanced the ability of the above two types of algal cells to absorb and enrich Cd^2+^.

The mechanism of heavy metal toxicity mainly involves [21,31]: (1) Binding to proteins or enzymes in organisms to inactivate them. (2) Interfering with metabolic processes such as respiration and photosynthesis. (3) Inducing oxidative stress. (4) Causing GSH depletion or antioxidant enzyme inhibition.

Parameters such as Fv/Fm and YII obtained by the chlorophyll fluorescence technique are the most useful parameters to assess the photosynthetic status of algae under heavy metal stress conditions [28]. This study showed that Cd^2+^ significantly reduced the maximum and actual photoconversion efficiency of *S. obliquus* and *C. pyrenoidosa* and the decrease was more pronounced with time. This inhibition was greatly improved by adding salicylic acid, suggesting that low concentrations of salicylic acid could counteract or keep the photosynthetic system of algal cells from Cd^2+^ attack. Scanning electron microscopy images of algal cells also reflected that Cd^2+^ made algal cells white, indicating that chloroplasts were damaged and photosynthetic reaction centers were inhibited.

The antioxidant enzymes of living organisms are fairly sensitive to pollutant stress and changes in their enzymatic activities can indirectly reflect the presence of oxidative contamination in the environment, thus serving as essential indicators for testing environmental pollution stress [32,33].

GSTs are a group of multifunctional enzymes. Their members vary in activity and function, some of which are essential for cell detoxification. They can form non-toxic derivatives by catalyzing the electrophilic group of harmful substances binding to the sulfhydryl group of reduced glutathione. Some GSTs also possess glutathione peroxidase activity, isomerase activity and thioltransferase activity. Therefore, in the face of adversity, such as heavy metals, organisms often rely on increased GST activity in their bodies for detoxification and anti-oxidation to stop the body from being damaged [34,35].

GSH-Px is an essential enzyme for the decomposition of peroxides that are widely present in the body. It can scavenge lipid hydroperoxides (LPO), remove H_2_O_2_ and mitigate the damage of organic hydroperoxides to the organism, such as catalyzing the decomposition of LPO to produce corresponding alcohols, preventing LPO homolysis and triggering the chain-branched reaction of lipid peroxidation to defend the organism from damage [36].

This study found that under Cd^2+^ stress, although the activities of both enzymes were elevated in the early stages in both *S. obliquus* and *C. pyrenoidosa*, the elevated activities of both enzymes lasted longer and were more significant after the addition of salicylic acid. Phenolic acids are known to provide antioxidant protection and adaptive responses to oxidative stress in eukaryotic algae due to their ability to provide electrons or hydrogen atoms at specific concentrations [37]. The security of algal cells by elevating GST and GSH-Px activity may be another mechanism of the low concentrations of salicylic acid.

## 5. Conclusions

(1) Salicylic acid can effectively inhibit cyanobacteria and relieve the stress of heavy metals in plants or algae. However, studies on adding salicylic acid under heavy metal stress have not been reported. The results of this study provide a new idea and experimental basis for the biological removal of heavy metal pollutants as well as heavy metal pollutants in eutrophic water.
(2) Cd^2+^ interacted with *S. obliquus* and *C. pyrenoidosa.* Cd^2+^ was toxic to both types of algae, but both species showed a good uptake and enrichment of Cd^2+^. The addition of salicylic acid at low concentrations (30–90 mg/L) counteracted or protected the algal cells from Cd^2+^ attack to varying degrees, increasing the biomass of both species and the uptake of Cd^2+^ by them.
(3) The mechanisms that the low concentration of salicylic acid counteracted or protected algal cells from Cd^2+^ attack, possibly related to the chelation of heavy metals by salicylic acid itself, which reduced the toxicity of Cd^2+^ and improved the photosynthetic efficiency of both *S. obliquus* and *C. pyrenoidosa*. In addition, salicylic acid promoted Cd^2+^ uptake in the above two species of green algae, probably due to the increased activity of GSH-Px and GST, which enhanced the antioxidant capacity of algal cells and maintained the relative integrity of algal cell morphology and structure, and also due to the chelation of the sulfhydryl group of GST with Cd^2+^, resulting in detoxification.
(4) In conclusion, eukaryotic algae have great potential for the adsorption and removal of heavy metals as an affordable material. An emerging area of research is the design and development of stronger algal strains, as traditional methods of using wild algae to reduce the concentrations of toxic metal ions are often costly. Molecular regulation of the levels of glutathione, lipopolysaccharide, phytochelin and metal thionin can improve the metal ion accumulation ability of algal cells; by supplementing phytohormones, such as salicylic acid, algae resistance to heavy metal toxicity can be increased. In addition, future research should have a set of rapid test methods to confirm various hypotheses as soon as possible, including the rapid determination of the chelating efficacy of each biomolecule chelator under the same pH, same temperature and same heavy metal concentration conditions.

## Figures and Tables

**Table 1:** Salicylic acid (mg/L) on the inhibitory rates (%) of *S. obliquus* and *C. pyrenoidosa* under Cd^2+^ stress.

Time (h)	*S. obliquus*					*C. pyrenoidosa*				
	ck2	30	60	90	120	ck2	30	60	90	120
24	17.5 ± 2.2 ^aA^	−7.8 ± 1.6 ^cA^	−1.6 ± 0.3 ^cA^	3.2 ± 0.5 ^bA^	22.7 ± 2.7 ^aA^	16.4 ± 2.3 ^aA^	3.1 ± 0.8 ^cA^	−11.9 ± 2.9 ^cA^	−1.7 ± 0.4 ^aA^	20.9 ± 2.7 ^aA^
48	38.0 ± 3.3 ^aB^	−3.3 ± 0.7 ^cB^	5.1 ± 1.4 ^bA^	34.7 ± 6.3 ^aB^	48.2 ± 3.5 ^aA^	33.7 ± 5.9 ^aB^	4.6 ± 1.1 ^cB^	9.1 ± 1.6 ^bC^	37.7 ± 4.9 ^aB^	47.4 ± 3.8 ^bAB^
72	58.2 ± 6.1 ^aC^	24.5 ± 2.9 ^bB^	20.4 ± 4.2 ^bC^	50.2 ± 7.1 ^aB^	65.6 ± 5.1 ^aB^	54.3 ± 4.6 ^aC^	21.0 ± 3.8 ^cC^	25.7 ± 2.9 ^bC^	53.2 ± 8.7 ^aB^	61.3 ± 4.6 ^bAB^
96	67.0 ± 7.5 ^aC^	17.5 ± 4.3 ^cC^	33.0 ± 5.8 ^bB^	55.3 ± 4.6 ^abB^	77.7 ± 6.2 ^aC^	61.4 ± 9.3 ^aC^	30.5 ± 4.9 ^cC^	28.5 ± 3.1 ^dB^	59.2 ± 6.5 ^bB^	71.3 ± 5.1 ^bB^

Note: Different lowercase letters indicate significant differences at the same time among each group; different capital letters indicate significant differences at different times in the same group.

## Data Availability

Not applicable.
